# Real-world effectiveness of hyperbaric oxygen therapy for delayed neuropsychiatric sequelae after carbon monoxide poisoning

**DOI:** 10.1038/s41598-021-98539-y

**Published:** 2021-09-28

**Authors:** Shu-Chen Liao, Shih-Chieh Shao, Kun-Ju Yang, Chen-Chang Yang

**Affiliations:** 1 Department of Emergency Medicine, Chang Gung Memorial Hospital, Keelung, Taiwan; 2 College of Medicine, Chang Gung University, Taoyuan, Taiwan; 3 School of Pharmacy, Institute of Clinical Pharmacy and Pharmaceutical Sciences, College of Medicine, National Cheng Kung University, Tainan, Taiwan; 4 Department of Pharmacy, Keelung Chang Gung Memorial Hospital, Keelung, Taiwan; 5 Division of Hyperbaric Oxygen Center, Chang Gung Memorial Hospital and Chang Gung University College of Medicine, Taoyuan, Taiwan; 6 Department of Emergency Medicine, Chang Gung Memorial Hospital, Linkou, Taiwan; 7 Institute of Environmental and Occupational Health Sciences, School of Medicine, National Yang Ming Chiao Tung University, Taipei, Taiwan; 8 Division of Clinical Toxicology & Occupational Medicine, Department of Medicine, Taipei Veterans General Hospital, 201 Shih-Pai Road Section 2, Taipei, 11217 Taiwan

**Keywords:** Neuroscience, Health care, Medical research, Neurology

## Abstract

To assess real-world effectiveness of hyperbaric oxygen therapy (HBOT) on delayed neuropsychiatric sequelae (DNS) after carbon monoxide (CO) poisoning we conducted a retrospective review of patients with CO poisoning admitted to Linkou Chang-Gung Memorial Hospital, Taiwan’s largest medical center, during 2009–2015. We included patients developing DNS after CO poisoning and compared improvements in neuropsychiatric function, with and without HBOT, after 12 months post-DNS to understand differences in recovery rates. DNS improvement-associated factors were also evaluated. We used receiver operating characteristic (ROC) curve analysis to assess the role of time elapsed between DNS diagnosis and HBOT initiation in predicting DNS improvement. A total of 62 patients developed DNS, of whom 11 recovered while the rest did not. Possible factors predicting DNS improvement included receiving HBOT post-DNS (72.7% vs 25.5%; P = 0.006), and treatment with more than three HBOT sessions during acute stage CO poisoning (81.8% vs 27.5%; P = 0.003). The relevant area under the ROC curve was 0.789 (95% CI 0.603–0.974), and the best cut-off point was 3 days post-DNS diagnosis, with 87.5% sensitivity and 61.5% specificity. Early HBOT in patients who developed DNS after CO poisoning significantly improved their DNS symptoms, with treatment effects sustained for 1 year after DNS diagnosis.

## Introduction

Carbon monoxide (CO) is one of the main environmental causes of acute poisoning worldwide, and is associated with a high mortality rate and specific complications^1–5^. CO often originates from the incomplete combustion of hydrocarbon fuels in fires, stoves, charcoal briquette burners, boilers, chimneys, internal combustion engines, gas-fuelled water heaters, and so on^2,5^. Most patients with CO poisoning present to the emergency department (ED) with nonspecific symptoms and fully recover after oxygen therapy. Long periods or high atmospheric concentrations of CO exposure however can cause damage to vital organs, which have high oxygen demand and consumption, such as the heart and the brain^6^. In addition to causing acute morbidity and mortality, CO poisoning can cause delayed neuropsychiatric sequelae (DNS), with the lag time ranging from 2 days to 6 weeks^7^. Since there are no strict diagnostic criteria, most reported DNS cover a broad spectrum of neurological deficits, including cognitive impairments, and behavioral and psychological disorders^8^. The outcome of DNS is relatively good in certain CO poisoned patients; in a study from Seoul, Korea, Choi et al. reported that three-fourths of patients with DNS recovered within 1 year^9^. The symptoms range from slight headache and anxiety to the most severe manifestations such as urinary/stool incontinence, inability to walk and dementia^8^. In severe cases, patients with DNS experience inability to independently ambulate and/or perform activities of daily living, with serious psychological, economic, and mental health consequences, which afflict the patients, their families, and the society^10^.

For the treatment of acute CO poisoning, hyperbaric oxygen therapy (HBOT) plays a certain role^7,11–13^. However, the treatment of DNS is still inconsistent and related literature is scant. Researchers have reported that delayed HBOT alone or in combination with ROS scavenger drugs had treatment effects on delayed encephalopathy^11,14–17^. The mechanisms of HBOT in DNS treatment remain unclear as well. HBOT may play a role in reducing oligodendrocyte damage, promoting precursor cell differentiation and remyelination of axons in deep white matter in cases of DNS following CO poisoning^18^. Also, some drugs have been used in DNS treatment. Zhang et al. reported *N*-butylphthalide and dexamethasone combined with HBOT significantly improved the cognitive and motor functions of CO poisoned patients, compared to HBOT alone. Originally isolated from celery seeds, *N*-butylphthalide soft capsules were launched in 2002 in China. *N*-Butylphthalide is a drug primarily used for cerebral arterial thrombosis in cerebrovascular disease, and its main effect is as a neuroprotective agent^6,10,19^. Xiang et al. reported a randomized trial with HBOT versus HBOT plus dexamethasone for DNS induced by CO poisoning; the results showed that the combination of dexamethasone and HBOT yielded better outcomes for patients with DNS^6,19^.

Current studies are still inconclusive with regard to the treatment of DNS, since most of the research is limited to case studies or local clinical practice in a certain area. An interventional study involving treatment with HBOT versus sham in a randomized crossover fashion is in progress in the USA. Since DNS causes significant suffering with numerous people in torment for months or even years, and HBOT is a relatively safe treatment with few side effects, we were curious about the therapeutic potential of HBOT after the development of DNS. Therefore, we conducted a retrospective study to investigate the role of HBOT in DNS treatment.

## Materials and methods

### Ethics

Chang Gung Medical Foundation’s Institutional Review Board (IRB) granted approval for this study protocol, and permission was given by the Medical Ethics Committee of Chang Gung Memorial Hospital under the number 104-7628C. Given the retrospective nature of this study, informed consent was waived by the above-mentioned committee.

### Study design, setting, population, and cohort selection

The study design involved retrospective review of the medical records of all patients admitted with CO poisoning to the emergency department (ED) of Linkou Chang Gung Memorial Hospital (CGMH), a tertiary medical center of approximately 3700 beds, between 1st Jan, 2009, and 31st Dec., 2015. Patients’ carboxyhemoglobin (COHb) levels were determined by arterial blood gas analysis, using a CO-oximeter. All enrolled patients had to have met the following criteria: first, the patient’s COHb level had to exceed 10% in smokers or 5% in non-smokers, as measured by the first intervening medical institution. Second, there had to be a witnessed CO exposure scenario (e.g., running an engine or burning charcoal in a confined, unventilated space, improper operation of a gas water heater or furnace, etc.). Finally, we only included those patients who developed DNS after CO poisoning.

### Data collection and definition of the variables

Data covering the following baseline characteristics were extracted from the medical records of all confirmed CO poisoning cases: age, gender, prior psychiatric history, exposure to CO intentionally or accidentally, CO source, initial vital signs at ED admission, concomitant alcohol- or tranquillizer use, loss of consciousness and duration thereof, time elapsed between CO exposure and admission to ED, treatment modality (HBOT at 2.5 ATA for 90 min with air/break 25/5 min or 100% NBO with ‘non-rebreathing’ facemask [NRM]), number of HBOT sessions in the acute poisoning and post-DNS stages, manifest symptoms of DNS (psychiatric, neurological or both), days from initial occurrence of DNS and duration from initial occurrence of DNS to recovery. All patients with a confirmed diagnosis of CO poisoning were given supplementary 100% oxygen using NRM upon ED admission. For patients with acute CO poisoning, HBOT was considered after consultation with an HBOT specialist in the study hospital and the treatment protocol followed the American Undersea and Hyperbaric Medical Society guidelines for HBOT in cases of CO poisoning (“Supplementary Materials S1” in Table S1). The HBOT specialist consultations were usually completed at the study hospital within 6 h after patients’ admission to the emergency department (ED) with suspected CO poisoning. However, the time elapsed between the onset of CO poisoning and the initiation of HBOT depended on the following factors: (1) the time elapsed between CO poisoning and ED visit; and (2) the availability of HBOT at the time of consultation. Our HBOT center is open all year round for emergency cases except for patients requiring ventilatory support, since a ventilator is not available inside the HBOT chamber. Those patients who received HBOT after DNS diagnosis all underwent HBOT as soon as the diagnosis of DNS was made. The treatment was conducted in accordance with the American Undersea and Hyperbaric Medical Society guidelines for HBOT in cases of CO poisoning (https://www.uhms.org/carbon-monoxide-poisoning/carbon-monoxide-poisoning.html).

Blood tests were checked on samples obtained at ED admission and during the course of the patients' hospital stay, which included complete blood cell count, arterial blood gas analysis, serum alcohol level, COHb level and troponin I and creatine phosphokinase levels. Urine samples were screened at admission for illicit drugs and benzodiazepines.

DNS are defined as new-onset or rapidly worsening neurological and/or psychiatric symptoms that develop within 2–42 days after CO poisoning, and may include unsteady gait, urinary incontinence, lethargy, emotional lability, difficulty in concentrating, mutism, apraxia, amnestic syndromes, Parkinsonism, cognitive impairment, dementia, etc. (“Supplementary Materials S1” in Table S2)^8^. All CO poisoned patients were invited to attend follow-up evaluation in outpatient clinics after hospital discharge, including comprehensive neurological- and mental status examination by a neurologist or a psychiatrist. The minimum follow-up period for the development of DNS was 6 months. EEG and/or brain imaging studies including CT scan and MRI were performed after the development of DNS, and additional HBOT sessions were considered for patients with DNS. When a patient subsequently developed DNS, the follow-up period was extended to 1 year. A patient was defined as having recovered from DNS if their daily activities returned to their baseline status before CO poisoning, based on an interview with the patient and patient’s family.

### Statistical analysis

Chi-square test or Fisher’s exact test was used to compare categorical variables of demographic and clinical data between patients with and without HBOT. DNS improvement within 12 months, and the associated factors were evaluated by univariate analyses. We compared the proportion of DNS improvement in patients with and without HBOT after 3, 6, 9 and 12 months post-DNS in order to understand the recovery rates at different times. We also used receiver operating characteristic (ROC) curve analysis to assess the role of the time elapsed between DNS diagnosis and initiation of HBOT in predicting DNS improvement. SAS Enterprise Guide, version 7.13 (SAS Inc., Chicago, IL, USA) was used for data analysis. A P-value < 0.05 was considered statistically significant.

## Results

**Figure 1:**
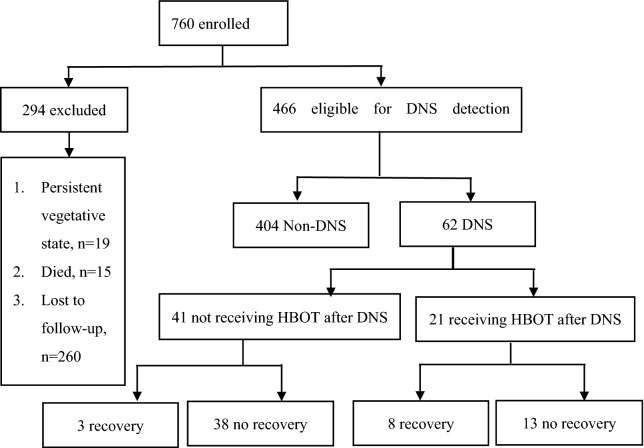
Study flowchart [Microsoft Word (v.2106, 2020, Office 365, Microsoft Corporation, Redmond, Washington, USA)].

We identified a total of 760 patients with CO poisoning who were admitted to the ED of Linkou Chang Gung Memorial Hospital from 2009 to 2015, and who met the inclusion criteria of this study. Among them, 466 patients were eligible for further DNS monitoring (Fig. 1). A total of 294 patients were excluded because we were unable to confirm or exclude the diagnosis of DNS. Among the excluded patients, 19 were in a persistent vegetative state or unresponsive wakefulness; 15 died during ED admission; and further contact was lost with 260 patients after discharge from the ED. Finally, we included 62 patients who developed DNS following CO poisoning. In the study cohort, slightly over half were men (35/62, 56.5%) and aged over 36 years old (34/62, 54.8%). We found the patients who received more than three HBOT sessions between CO poisoning and the onset of DNS (76.2% vs 17.1%; P < 0.0001) and those with early occurrence of DNS following the CO poisoning (≤ 7 days: 52.4% vs 17.1%; P = 0.003) were more likely to receive HBOT after the development of DNS. The interval between DNS development and the initiation of HBOT for DNS treatment varied from 0 to 39 days because not all patients with DNS sought medical attention immediately after developing clinical manifestations of DNS, such as memory impairment, concentration deficit, ataxia and incontinence. Eight patients received HBOT on the same day of their DNS development, four patients within 1–3 days, five patients within 3–5 days, two within 2–10 days, and three within 10–39 days after the onset of DNS. Other demographic information of patients with and without HBOT therapy after DNS development is presented in Table 1.

**Table 1:** Demographic and clinical data of patients with and without HBOT after DNS development.

Variables	HBOT (*n* = 21)	No HBOT (*n* = 41)	*P* value
**Age**			0.058
<= 36 years old	13 (61.9%)	15 (36.6%)	
> 36 years old	8 (38.1%)	26 (63.4%)	
**Gender**			0.937
Male	12 (57.1%)	23 (56.1%)	
Female	9 (42.9%)	18 (43.9%)	
**Receipt of HBOT at acute poisoning stage**			
Yes	19 (90.5%)	28 (68.3%)	0.054
No	2 (9.5%)	13 (31.7%)	
**Number of HBOT sessions at acute poisoning stage**			
< =3	5 (23.8%)	34 (82.9%)	< 0.0001
> 3	16 (76.2%)	7(17.1%)	
**Intentional poisoning**			
Yes	17 (81.0%)	25 (61.0%)	0.111
No	4 (19.1%)	16 (39.0%)	
**Source of CO poisoning**			0.290
Charcoal burning	17 (81.0%)	28 (68.3%)	
Not charcoal burning	4 (19.0%)	13(31.7%)	
**Transient loss of consciousness at acute poisoning stage**			0.406
Yes	20 (95.2%)	35 (85.4%)	
No	1 (4.8%)	6 (14.6%)	
**GCS less than 9 at ED**			0.743
Yes	7 (33.3%)	12 (29.3%)	
No	14 (66.7%)	29 (70.7%)	
**Intubation during hospitalization**			0.735
Yes	3 (14.3%)	8 (19.5%)	
No	18 (85.7%)	33 (80.5%)	
**Concomitant alcohol drinking**			1.000
Yes	2 (9.5%)	3 (7.3%)	
No	19(90.5%)	38 (92.7%)	
**Concomitant use of tranquilizer**			0.565
Yes	6 (28.6%)	9 (22.0%)	
No	15 (71.4%)	32 (78.0%)	
**Any complication**			
Yes	11 (52.4%)	20 (48.8%)	0.788
No	10 (47.6%)	21 (51.2%)	
**Symptoms of DNS**			0.178
Both neurological and psychiatric	10 (47.6%)	10 (24.4%)	
Neurological	5 (23.8%)	15 (36.6%)	
Psychiatric	6 (28.6%)	16 (39.0%)	
**Days from CO exposure to DNS**			0.003
< =7	11 (52.4%)	7 (17.1%)	
8–14	1 (4.8%)	14 (34.2%)	
15–21	0 (0%)	6 (14.6%)	
> 21	9 (42.9%)	14 (34.2%)	

Continuous data are expressed as mean ± standard deviation; categorical data are presented as frequency (proportion).

*HBOT* hyperbaric oxygen therapy, *DNS* delayed neuropsychiatric sequelae, *GCS* Glasgow Coma Scale, *ED* emergency department.

**Figure 2:**
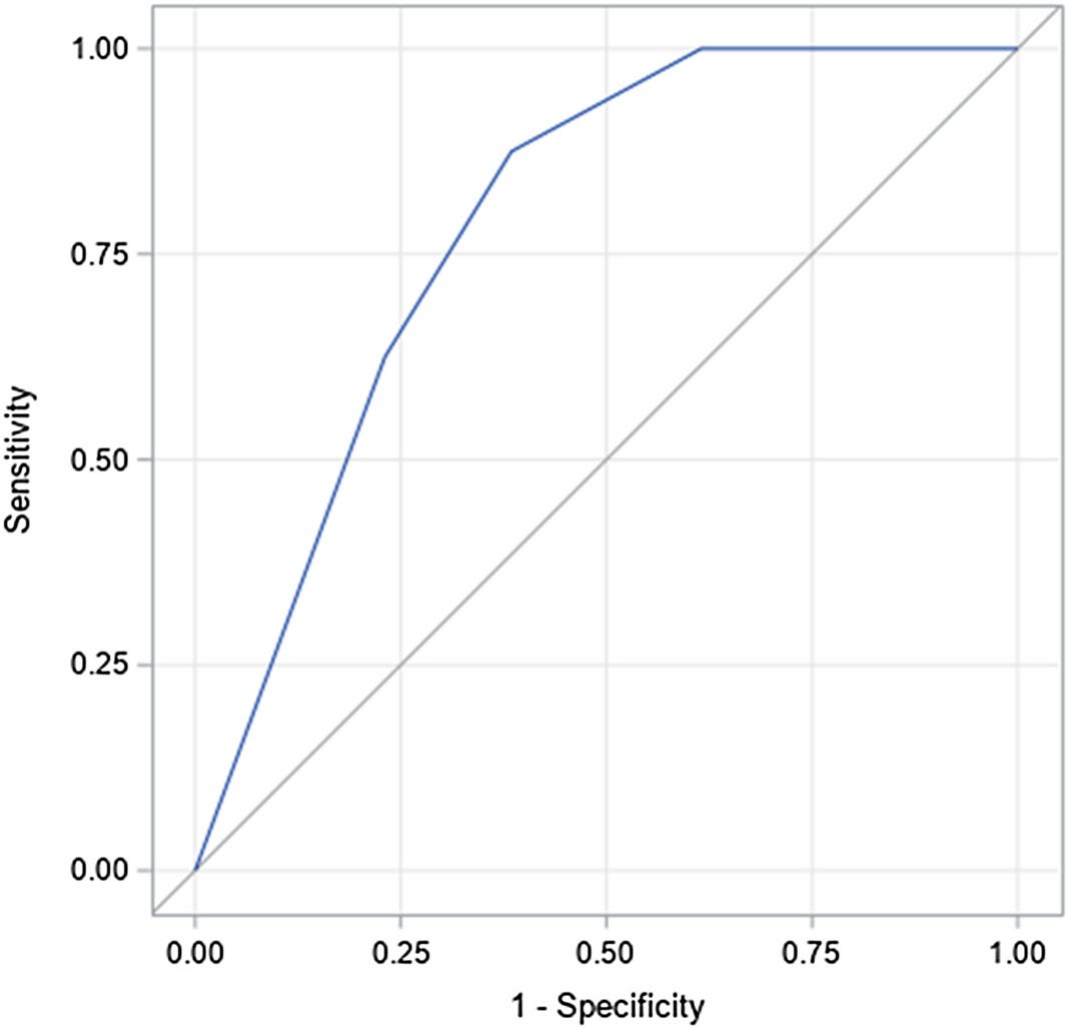
ROC curve for the duration from DNS diagnosis to the initiation of hyperbaric oxygen therapy for assessing the recovery in patients who received hyperbaric oxygen therapy. The area under the ROC curve is 0.789 (95% CI 0.603–0.974). The best cut-off point is at 3 days with a sensitivity of 0.875 and a specificity of 0.615. [SAS Enterprise Guide, version 7.1 (SAS Institute, Cary, NC, USA)].

Univariate analysis revealed that possible factors associated with DNS improvement included receiving HBOT after the development of DNS (72.7% vs 25.5%; P = 0.006) and treatment with more than three HBOT sessions between CO poisoning and the onset of DNS (81.8% vs 27.5%; P = 0.003) (Table 2). Specifically, we found that patients who received HBOT at an earlier time post-DNS experienced a significant and faster improvement in their DNS symptoms, and the treatment effects were sustained for 1 year after the diagnosis of DNS (Table 3). Figure 2 plots the ROC curve for the duration from DNS diagnosis to initiation of HBOT in order to assess the recovery in patients who received HBOT post-DNS. The relevant AUC was 0.789 (95% CI 0.603–0.974), and the best cut-off point was 3 days post-DNS diagnosis, with a sensitivity of 87.5% and a specificity of 61.5%.

**Table 2:** Factors associated with the recovery from DNS.

Variables	No recovery (N = 51)	Recovery (N = 11)	*P* value
**Receipt of HBOT after DNS**			0.006
Yes	13 (25.5%)	8 (72.7%)	
No	38 (74.5%)	3 (27.3%)	
**Age**			0.183
<= 36 years old	21 (41.2%)	7 (63.6%)	
> 36 years old	30 (58.8%)	4 (36.4%)	
**Gender**			0.239
Male	27 (52.9%)	8 (72.7%)	
Female	24 (47.1%)	3 (27.3%)	
**Receipt of HBOT at acute poisoning stage**			0.962
Yes	36 (70.6%)	11(100%)	
No	15 (29.4%)	0 (0%)	
**Number of HBOT sessions at acute poisoning stage**			0.003
< =3	37 (72.5%)	2 (18.2%)	
> 3	14 (27.5%)	9 (81.8%)	
**Source of CO poisoning**			0.466
Charcoal burning	38 (74.5%)	7 (63.6%)	
Not charcoal burning	13 (27.5%)	4 (36.4%)	
**Transient loss of consciousness at acute poisoning stage**			0.800
Yes	45 (88.2%)	10 (90.9%)	
No	6 (11.8%)	1 (9.1%)	
**GCS less than 9 at ED**			0.119
Yes	18 (35.3%)	1 (9.1%)	
No	33 (64.7%)	1 (90.9%)	
**Intubation during hospitalization**			0.950
Yes	11 (21.6%)	0 (0%)	
No	40 (78.4%)	11 (100%)	
**Concomitant alcohol drinking**			0.967
Yes	5 (9.8%)	0 (0%)	
No	46 (90.2%)	11 (100%)	
**Concomitant use of tranquilizer**			0.610
Yes	13 (25.5%)	2 (18.2%)	
No	38 (74.5%)	9 (81.8%)	
**Any complication**			0.739
Yes	26 (51.0%)	5 (45.5%)	
No	25 (49.0%)	6 (54.5%)	
**Symptoms of DNS**			0.338
Both neurological and psychiatric	17 (33.3%)	3 (27.3%)	
Neurological	18 (35.3%)	2 (18.2%)	
Psychiatric	16 (31.4%)	6 (54.5%)	
**Days from CO exposure to DNS**			0.930
<= 7	14 (27.5%)	4 (36.4%)	
8–14	13 (25.5%)	2 (18.2%)	
15–21	5 (9.8%)	1 (9.1%)	
> 21	19 (37.3%)	4 (36.4%)	

Continuous data are expressed as mean ± standard deviation; categorical data are presented as frequency (proportion).

*HBOT* hyperbaric oxygen therapy, *DNS* delayed neuropsychiatric sequelae, *GCS* Glasgow Coma Scale, *ED* emergency department.

**Table 3:** Recovery time for patients with and without HBOT after DNS development.

Recovery time, n	No HBOT (n = 41)	HBOT (n = 21)	*P* value
Within 3 months	2 (9.5%)	7 (17.1%)	0.005
Within 6 months	2 (9.5%)	8 (19.5%)	0.002
Within 9 months	3 (14.3%)	8 (19.5%)	0.005
Within 12 months	3 (14.3%)	8 (19.5%)	0.005

Categorical data are presented as frequency (proportion).

*HBOT* hyperbaric oxygen therapy, *DNS* delayed neuropsychiatric sequelae.

## Discussion

Our study demonstrated that an improvement in the neuropsychiatric sequelae was more commonly observed in those patients who received HBOT after DNS development and in those who received more than three sessions of HBOT at the acute poisoning stage. Moreover, the initiation of HBOT within as little as three days in DNS patients post-diagnosis produced significant improvement in DNS symptoms, and the treatment effects were sustained for 1 year after the diagnosis of DNS.

Researchers have made little remarkable progress in the treatment of DNS. While over half of DNS patients may recover spontaneously within 1 year^1^; once they develop DNS, rehabilitation arrangements and symptomatic treatment with medications remain the primary therapeutic options. The pathophysiology of DNS after CO poisoning is unclear, but most researchers consider it to be related to demyelination, destruction and apoptosis of neurons in cerebral white matter and the globus pallidus. Inflammation caused by CO poisoning also plays a role in DNS development, including cytokine release, lipid peroxidation, mitochondrial oxidative stress, inhibition of mitochondrial function, and adaptive immunological responses, all of which influence the pathogenesis of neuron damage^13^.

Some medicines have been reported to be effective in the treatment of DNS. Huarcaya-Victoria et al. reported a 37-year-old woman who recovered from her psychotic and catatonic symptoms after treatment with risperidone 4 weeks after CO poisoning^20^. Song et al. reported a 16-year-old man who suffered acute CO poisoning and benefited from donepezil hydrochloride treatment. The young man presented to the ED with stupor consciousness after CO poisoning, but he totally recovered 2 days later after acute resuscitation. DNS developed on the 25th day after acute poisoning; both risperidone and donepezil hydrochloride were prescribed to manage neurologic and psychiatric deficits. The patient recovered from cognitive impairment but there was no improvement of his gait dysfunction. Traditionally, donepezil hydrochloride is indicated for the symptomatic treatment of mild to moderately severe Alzheimer's dementia. Acetylcholine is a neurotransmitter in the brain that is important in memory function. Donepezil hydrochloride acts as a specific and reversible inhibitor of acetylcholinesterase, and is thought to prevent the breakdown of acetylcholine to preserve brain function^21,22^. Yanagiha et al. reported two cases treated with HBOT plus donepezil hydrochloride, which alleviated delayed cognitive impairment caused by CO poisoning^22^.

Xiang et al. conducted a randomized controlled trial to evaluate the treatment effect of HBOT versus HBOT plus dexamethasone. They found that for patients with DNS, the combination treatment of dexamethasone and HBOT for 4 weeks was more effective than HBOT alone, and yielded a better index of cognitive function assessed by Mini-Mental State Examination (MMSE), and a lower level of myelin basic protein (MBP), a CSF biomarker of DNS^23^. Inflammation plays a crucial role in DNS induced by acute CO poisoning, and dexamethasone may play a role by protecting myelin from damage as a result of inflammatory response attributable to brain tissue lipid peroxidation^23,24^. Spina et al. reported a 33-year-old woman suffering from DNS 40 days after accidental CO poisoning. DNS was treated successfully with HBOT and concurrent reactive oxygen species (ROS) scavenger, i.e., high dose of *N*-acetylcysteine (NAC, 12 g/day in continuous infusion) and low dosage of oral glucocorticoids (prednisone 25 mg/day)^25^. HBOT might either increase systemic oxidative stress or cause oxygen free radical overproduction, and ROS are known to mediate O_2_ toxicity. Both glucocorticoids and NAC have synergistic effects to offset the supposed physiopathology of DNS and the presumptive oxidative stress induced by HBOT. A combination of HBOT and antioxidant as well as anti-inflammatory drugs may therefore produce a positive synergistic effect on DNS treatment. Xiang et al. conducted a randomized trial comparing HBOT only and HBOT plus *N*-butylphthalide, a chemical constituent of celery oil, to evaluate DNS treatment efficacy. They found the combined application of *N*-butylphthalide and HBOT could be a potentially effective therapy in treating cognitive dysfunction in patients with DNS. Studies in animal models suggest that butylphthalide may be useful for the treatment of hypertension and may have neuroprotective effects. *N*-Butylphthalide could improve energy metabolism of the brain, increase microcirculation in the ischemic brain regions, and thus result in improved memory- and cognitive function^6,26,27^.

There is a formal treatment protocol for acute CO poisoning in our hospital. We perform HBOT at a pressure of 2.5 ATA, with 100% oxygen concentration, for 90 min duration with air/break for 25/5 min, and a treatment interval of once a day for 3 consecutive days, leading to a total of three sessions of HBOT. However, some patients do not receive three sessions of HBOT at the acute stage of CO poisoning because in general, only inpatients are eligible to receive three sessions of HBOT that are then reimbursed by Taiwan’s National Health Insurance, and some patients refuse to be hospitalized and may decline self-paid HBOT.

As for the treatment of DNS, currently there is no formal HBOT protocol, in terms of the number of treatment sessions, at the study hospital. The number of sessions varied from 1 to 30 sessions in this study, with a median of six sessions. Nevertheless, the treatment pressure, oxygen concentration, duration and air/break time, and the interval between two sessions were the same as those for the treatment of acute CO poisoning. In this study, we found HBOT to be an effective treatment after the development of DNS (HBOT vs non-HBOT, 61.9% vs 38.1%; P = 0.006). HBOT administered after the diagnosis of DNS can accelerate patients’ recovery, avoiding waiting for the natural recovery process or going through a long rehabilitation course. Early HBOT intervention within 3 days after the development of DNS seems beneficial to the patient’s outcome.

The mechanism of recovery through HBOT after the development of DNS may be related to the transfer of functional mitochondria to the injury site, re-myelination of damaged neurons, angiogenesis and neurogenesis, production of anti-inflammatory cytokines and balancing inflammatory and anti-inflammatory cytokines to protect the integrity of the blood–brain barrier^28–30^. The benefit of HBOT in the treatment of DNS after acute CO poisoning has been debated for decades. A retrospective cohort study of small sample size indicated that HBOT was effective for DNS treatment after a longer interval following acute CO poisoning (30–60 days)^15^. Those patients all suffered from both neurologic and psychiatric sequelae after they recovered from acute CO poisoning. All patients received EEG, MMSE, brain MRI, event-related potential (ERP) and brain perfusion scan (brain SPECT) before and after the initiation of HBOT. After 8–40 sessions of HBOT, functional neurological tests including ERP, EEG, MMSE, and brain SPECT showed improvements that were compatible with the patients’ clinical presentations^15^. As reported in previous studies, CO caused platelets to release NO, an agent known to inhibit the function of β2 integrins. In vitro studies have demonstrated that β2 integrin-dependent polymorphonuclear leukocyte adherence was impaired immediately following CO poisoning^31^. Compared with the experimental group (i.e., CO poisoning group, in vivo animal study), the control group (sham-treated animals) did not present with the conversion reaction of xanthine dehydrogenase (XD) to sulfhydryl-irreversible xanthine oxidase (XO), and subsequent lipid peroxidation. The results suggest that leukocytes are responsible for the development of biochemical changes in the brain, following CO poisoning, with the sequence of events occurring as follows: leukocyte sequestration in the microvasculature, β2 integrin-dependent adherence, and protease-mediated conversion of XD to XO, ultimately leading to O_2_ radical-dependent brain lipid peroxidation, which is thought to be the underlying process responsible for the clinical syndrome of DNS^32,33^. The proposed mechanism of HBOT in treating DNS is that HBOT could improve post-ischemic or inflammatory tissue survival by increasing reactive species to temporarily inhibit β2-integrin function of neutrophils, as well as inducing antioxidant enzymes and anti-inflammatory proteins in many tissues^15^.

Several investigators have reported a markedly improved neuropsychiatric status among patients with DNS who received HBOT after having the sequelae. These patients developed DNS weeks to months after acute CO poisoning, and all presented to the hospital with severe cognitive and neurologic deficits that cost them independence in daily life activities. They underwent 10–40 sessions of HBOT, whereby periodic assessments by neuropsychologic test and image study were done; in some of the patients it was found that the lesions over the globus pallidus and white matter had disappeared after HBOT, which was also consistent with their symptoms^16,25,34,35^.

HBOT offers a treatment possibility for some cerebrovascular diseases, attenuating any inflammation process, such as traumatic brain injury (TBI) and ischemic stroke. Studies have revealed that HBOT can transfer functional mitochondria to the injury site, and attenuate the inflammation process by reducing cytokines crossing the blood–brain barrier, which plays a role in the secondary cell injury mechanism^36^. In an animal study, HBOT was provided 3 days after inducing a TBI model; HBOT significantly reduced TBI-induced motor and cognitive dysfunction by stimulating angiogenesis, neurogenesis and massive production of an anti-inflammatory cytokine: IL-10^28^. A distinct increase in basic protein isoforms of myelin following HBOT was also confirmed in another TBI animal study. Moreover, it has been suggested that the long-term protective effects of HBOT following brain injury might be catalyzed by a significant re-myelination in the injured ipsilateral cortex, as evidenced by the associated recovery of sensorimotor function^29^.

Some studies have shown that HBOT can improve cerebral blood circulation through non-recoverable ischemic regions of the brain and enhance neuroplasticity, with subsequent cognitive improvements in human subjects. HBOT can be applied in the treatment of dementia; and in animal studies, HBOT has induced angiogenesis by upregulating hypoxia-inducible factor-1*α* and vascular endothelial growth factor, even in the hippocampus^37^. A higher number of HBOT sessions has been shown to boost the vascular density within the hippocampus and upgrade spatial learning in animals; in adult rats with vascular dementia, more HBOT treatment has also been shown to shrink the area of cortical infarct, and enhance cerebral blood flow^38^.

The other possible predictor of DNS improvement in our study is receiving more than three sessions of HBOT at the acute poisoning stage. In the randomized controlled trial conducted by Weaver et al., three standard sessions of HBOT were arranged after CO poisoning, which were found to be effective against the development of DNS^39^. However, Carstairs et al. conducted a study in a murine model focusing on the number of HBOT sessions after CO poisoning, and found that there was no difference between groups treated with either one or three HBOT sessions^40^. Only a few literature reports have focused on how many sessions of HBOT are appropriate at the acute CO poisoning stage to prevent DNS, and a range from one session to 40 or more sessions have been reported in small sample-size prospective studies or case reports^15,16,25,34^. Further larger scale studies are warranted to clarify the optimal treatment sessions at the acute CO poisoning stage for DNS prevention.

There are several limitations to this study. First, the number of follow-up visits by patients after ED discharge declined steadily, reducing the ability to detect DNS in all CO poisoned patients and adversely affecting the study’s statistical power. Second, because of the lack of standardized diagnostic criteria for DNS, we adopted several symptom-based criteria. Previous studies have deployed test bundles as the diagnostic criteria for DNS^39^; however, these tests have remained inconclusive. Such test bundles have included the Folstein Mini-Mental Status Examination (MMSE), the Hamilton Depression Rating Scale (HDRS), Beck Anxiety Inventory, Wechsler Memory Scale-Revised (WMS-R) and Verbal Memory Process Test (VMPT)^41,42^. That notwithstanding, not every patient in this study received imaging study or the bundled tests on follow-up visits. Third, recovery from DNS was defined as the patient having regained the baseline state of their daily life activities prior to the CO poisoning event, which was assessed by interviewing the patient’s family. Because the baseline neuropsychiatric status of patients before CO exposure was unknown in certain patients, we were unable to completely exclude the possibility of pre-existing dementia and/or other neuropsychiatric disorders that might influence the development of DNS. Given that the majority of patients included in this study were young and had no history of pre-existing neuropsychiatric diseases, the above-mentioned possibility of information bias was likely to be low.

Fourth, since our HBOT facility cannot accommodate patients under mechanical ventilatory support, those requiring mechanical ventilation were excluded from this study. Fifth, when identifying a possible predictor for the outcome of study interest, it has been suggested that events per variable of ten or more should be available to avoid the overfitting problem. Since we only identified eight cases recovering from DNS after CO poisoning in this study, the possible predictors of DNS recovery could only be determined by univariate analysis. Finally, given that this was a retrospective study for which data were extracted by chart review, incomplete documentation of clinical presentations in the medical records might have compromised the identification of some potential significant predictors of DNS recovery among CO poisoned patients who were treated with HBOT.

## Conclusions

There is, at present, still no consensus about DNS treatment. Our study found that among patients who developed DNS, the group of those receiving more than three sessions of HBOT in the acute poisoning stage had a better prognosis with regard to subsequent neuropsychiatric sequelae than the other group of patients. In addition, after the development of DNS, the receipt of HBOT, especially the initiation of treatment within three days after the onset of DNS, seems to be associated with significantly improved their DNS symptoms.

## Supplementary Information

Supplementary Tables.

## Data Availability

The datasets generated during and/or analysed during the current study are available in the Figshare repository (accession number: 10.6084/m9.figshare.15000753).
