# Supplementary material for: Real-world effectiveness of hyperbaric oxygen therapy for delayed neuropsychiatric sequelae after carbon monoxide poisoning
Source: Sci Rep. 2021 Sep 28;11:19212. doi: 10.1038/s41598-021-98539-y (PMC8479087; doi:10.1038/s41598-021-98539-y)
Supplement: Supplementary file 1 — Supplementary Tables. [file 41598_2021_98539_MOESM1_ESM.docx]

**Supplementary Tables for the Article:**

Real-world effectiveness of hyperbaric oxygen therapy for delayed neuropsychiatric sequelae after carbon monoxide poisoning

Shu-Chen Liao^,^, Shih-Chieh Shao, Kun-Ju Yang, Chen-Chang Yang

**Table S1**. Hyperbaric oxygen therapy (HBOT) indications for CO poisoning and relevant treatment protocol in Chang-Gang Memorial Hospital (CGMH)

| 1. HBOT indicated for CO poisoning 2. Loss of consciousness 3. Ischemic cardiac changes 4. Neurological deficits 5. Significant metabolic acidosis 6. Carboxyhemoglobin ≧25% 7. Pregnant women 8. HBOT treatment protocol in CGMH 9. Patients receive treatment at 2.5 atmospheres absolute pressure for 90 minutes with 25/5 mins air-break. 10. Frequency: once daily 11. Total number of sessions suggested by hyperbaric oxygen specialist: ≧3 times 12. Oxygen supplementation route in HBOT chamber: face mask |
| --- |

Adopted from “Practice recommendations in the diagnosis, management, and prevention of carbon monoxide poisoning,” by Neil B. Hampson, Claude A. Piantadosi, Stephen R. Thom, and Lindell K. Weaver Am. J Respir Crit Care Med, 2012;186:1095–1101, Copyright 2012 by the American Thoracic Society.

**Table S2.** Previously reported signs and symptoms of delayed neuropsychiatric sequelae (DNS)

| **Neurological sequelae** | **Cognitive and psychological sequelae** |
| --- | --- |
| Parkinson-like syndromes | Concentration deficit |
| Gait and motor disturbances | Memory loss |
| Bradykinesia | Cognitive impairment |
| Intention tremor | Dementia |
| Myoclonus | Personality changes |
| Dyspraxia | Anxiety |
| Dysphasia | Extreme emotional lability |
| Ataxia | Psychosis |
| Postural instability | Depression |
| Vertigo | Mania |
| Cortical blindness | Insomnia |
| Hearing loss, tinnitus |  |
| Chorea |  |
| EEG abnormalities |  |
| Epilepsy |  |
| Peripheral neuropathies |  |
| Recurrent headache |  |
| Fecal/urinary incontinence |  |

Adopted from “Delayed neuropsychological sequelae after carbon monoxide poisoning: predictive risk factors in the emergency department. A retrospective study” by Giuseppe Pepe, Matteo Castelli, Peiman Nazerian, Simone Vanni, Massimo Del Panta, Francesco Gambassi, et al., Scand J Trauma Resusc Emerg Med, 2011;19:16.
